# A computer-controlled multi-electrode switch

**DOI:** 10.1155/S1463924699000164

**Published:** 1999

**Authors:** Fernando J. V. Santos, M. Soledade C. S. Santos

**Affiliations:** 1 Departamento de Química e Bioquímica, CCMM Faculdade de Ciências Universidade de Lisboa Campo Grande Lisboa 1749-016 Portugal; 2 CECUL, Faculdade de Ciências Universidade de Lisboa Campo Grande Lisboa 1749-016 Portugal

## Abstract

A computer-actuated switch was built to control, simultaneously, two automatic titration assemblies each consisting of an electrode pair and a burette, and using only one measuring device. This switch is modular, simple and versatile allowing easy adaptation and expansion; apart from its application in multiple-titration systems, this device can also be used for standard addition analysis and multi-component analysis using ion-selective electrodes (ISE). The repeatability as well as the accuracy of the measurements made with this switch were ensured using high-quality relays, and very high electrical insulation, attained through the use of two separate printed circuit boards (pcb) of good quality and careful design of these pcbs. This low-cost multi-electrode switch is controlled through the parallel port of a PC that collects the data via an inexpensive 12-bit ADC board (8-bit ISA type), and is easily programmable in any high-level language. This type of device allows the collection of a large amount of data in relatively short periods, which can be analysed later allowing the choice of the best compromise of time versus accuracy for the study of any particular system.


Journal of Automated Methods & Management in Chemistry, Vol. 21, No. 4 (July–August 1999) pp. 135–138

### A computer-controlled multi-electrode switch

**Fernando J. V. Santos and**
**M. Soledade C. S. Santos**[1]

*Departamento de Química e Bioquímica, CCMM, e-mail: fernando.santos@fc.ul.pt; [1]CECUL, Faculdade de Ciências, Universidade de Lisboa, Campo Grande, 1749-016 Lisboa, Portugal*

*A computer-actuated switch was built to control, simultaneously, two automatic titration assemblies each consisting of an electrode pair and a burette, and using only one measuring device. This switch is modular, simple and versatile allowing easy adaptation and expansion; apart from its application in multiple-titration systems, this device can also be used for standard addition analysis and multi-component analysis using ion-selective electrodes (ISE). The repeatability as well as the accuracy of the measurements made with this switch were ensured using high-quality relays, and very high electrical insulation, attained through the use of two separate printed circuit boards (pcb) of good quality and careful design of these pcbs. This low-cost multi-electrode switch is controlled through the parallel port of a PC that collects the data via an inexpensive 12-bit ADC board (8-bit ISA type), and is easily programmable in any high-level language. This type of device allows the collection of a large amount of data in relatively short periods, which can be analysed later allowing the choice of the best compromise of time versus accuracy for the study of any particular system.*

### Introduction

The use of chemical sensors in quantitative analysis has received a growing amount of attention over the last three decades. In particular, multi-component analysis using ion-selective electrodes (ISE) as chemical sensors is a rapidly expanding field [1–7]. The increasing number of selective electrodes available, and currently under development, has allowed the establishment of new rapid and accurate analytical procedures, in particular in the areas of clinical and pharmaceutical analysis.

The desirable sensitivity, accuracy and cost usually condition the choice of the measurement technique among direct potentiometry, standard addition or titrimetric determinations [1, 2, 4, 8]. However, the facility of access to microcomputers has revolutionized chemical laboratories, and resorting to automation may significantly improve the convenience and/or accuracy of all these techniques.

Direct potentiometry is the least accurate procedure, and despite its simplicity, both in terms of accuracy and automation, will probably tend to be substituted by more accurate and demanding approaches, e.g. chemometric analysis, standard addition or titrimetric determinations. The use of these techniques markedly improves the attainable analytical precision, usually at the expense of time and convenience, but the rapidly increasing capabilities of automation and mathematical analysis of microcomputers are likely to overcome these drawbacks in the near future.

The optimization of ISE, under development, usually aims for stability of response at the expense of some decrease in the selectivity of the sensor's response, a limitation that can be greatly improved by the appliance of multi-component chemometric analysis [1, 2, 4, 6]. Automation for this type of approach can be accomplished by accurate and versatile electrode switches, but subsequent data analysis, using non-linear regression techniques, is a process dependent on user-defined parameters thus difficult to automate.

Accuracy improvements by means of standard additions and titrimetric procedures on the other hand involve burette control apart from electrode switching and data acquisition capabilities, and therefore comprise a more laborious construction. Nonetheless, there are, nowadays, several standard addition and titrimetric apparatus commercially available, although most of them are rather complex and expensive 'black boxes' designed to meet the needs of routine analysis, for pre-set standard methods industrially accepted, with limited versatility [3, 6–9].

In the analysis of amine solutions, containing silver, in organic media, we faced several interferences, so in order to monitor both concentrations, independently and simultaneously, we decided to construct an inexpensive but accurate double titration assembly [10, 11].

This paper describes the computer-controlled double electrode pair–burette switch constructed and used to select the pair of working electrodes, as well as the titrant-delivering burette associated with it. This device can be attached to any pH meter in conjunction with any automatic burettes, and can also be used exclusively as a multi-electrode switch, apart from being modular, and consequently, easily replicated.

### Instrumentation

The double titration system constructed is schematically represented in figure 1, and consists of two mechanical piston-type burettes, Radiometer model ABU 12 and two Radiometer microelectrodes pairs (glass G2040C, calomel K4040 and silver P4040, calomel K6040); a PC-type microcomputer, Commodore 10 MHz 8088, with a 12-bit ADC add-on board, F.C.L. Dep. Física model ADC8x12, the double electrode pair–burette switch, and a pH meter, Radiometer model PHM 64 (0–1500 mV, 0.1 mV resolution and input impedance $> 10^{12}\,\Omega$).

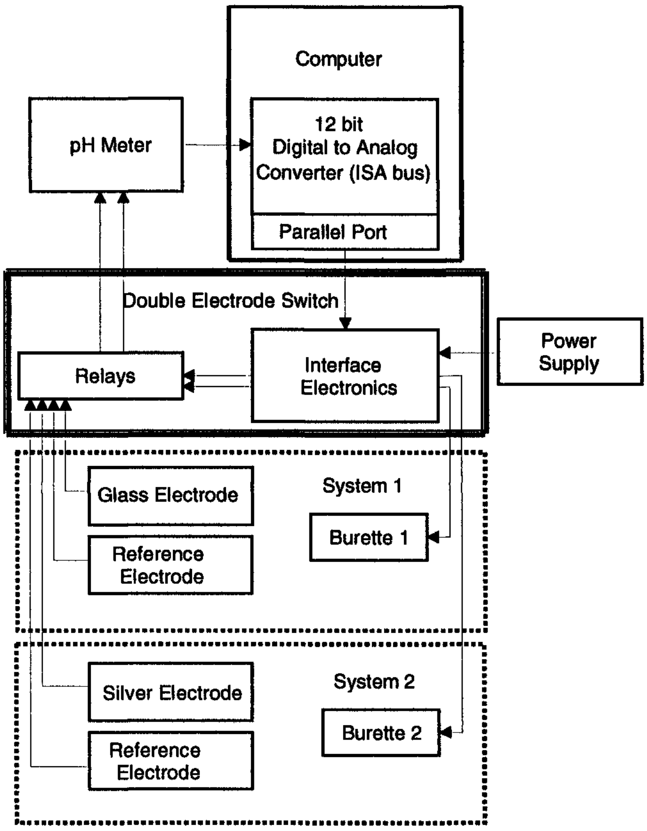

*Figure 1. Block diagram of the double titration system evidencing the multi-electrode switch.*

The PC commands the electrode switch through the standard parallel port (LPT1) and collects the experimental data, available at the analogue output (0–5 V) of the pH meter, through the ADC board. The ADC board used is an 8-bit ISA plug-in with eight differential input channels of 12 bit resolution, 25 ms conversion time, input impedance $> 10^9\,\Omega$ and a resolution of 0.25 mV. This add-on board was built in the Physics Department of our University, but similar boards are commercially available from Keithley, National Instruments, Omega, Data Translation, as well as other major hardware manufacturers.

External devices, e.g. the electrode switch, are easily actuated by a computer through the use of one of the available computer ports, either the parallel or serial type. For a small number of devices, the parallel port offers eight data lines and several control lines as direct output lines that can be used to directly feed relays, lamps, motors or any other electrical devices of compatible power requirements. The serial port, on the other hand, only provides one line for direct control of external devices, requiring the introduction of interface circuits to allow the control of more than one device. For devices requiring more power than the one available at the computer ports, the insertion of an external power supply and Darlington-type insulation between the computer electrical line and the device supply allows the accomplishment of computer command.

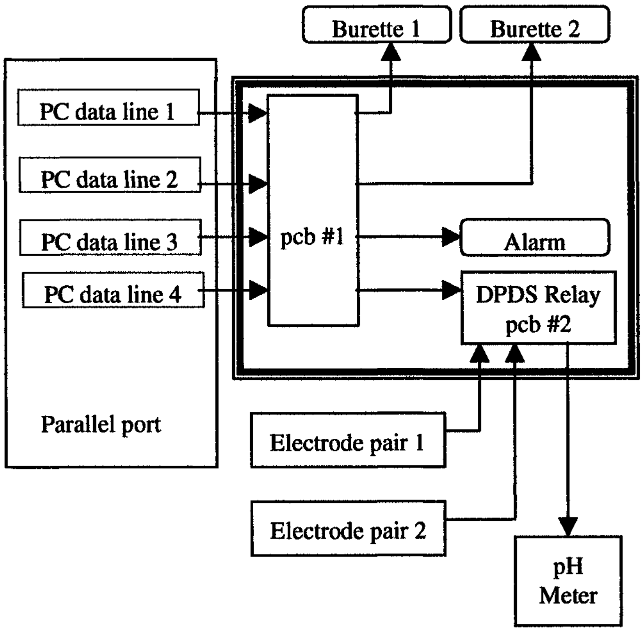

*Figure 2. Block diagram of the double electrode switch and connections.*

Apart from the previously mentioned advantages, the control software of the parallel port is straightforward and composed of just one command line for each order, either ON or OFF, for each of the devices being controlled: basically an 'OUT Port Address, Value' command, e.g. 'OUT &H378, 2' that can be implemented in almost any programming language. Simultaneous actuation of more than one device is also possible by just sending the value corresponding to the sum of the different devices control numbers. On the contrary, the use of a serial port is not as simple and always requires the definition of a transmission protocol besides the opening of the port for I/O prior to the execution of the first command instructions.

The experimental requirements for the particular case which leads to the construction of such a double electrode switch, set the necessities for simultaneous but independent control of each burette and electrode pair system. Such prerequisites lead to the choice of the parallel port as the most straightforward approach to command the electrode switch and burettes.

The double electrode switch built is composed of two independent printed circuit boards (pcb), as shown in figure 2, where two burettes, two electrode pairs and one alarm are controlled by the computer.

Two different pcbs were constructed in the laboratory, i.e. pcb#1 and pcb#2, on RS Components single-sided copper-clad epoxy glass board that were tin-plated afterwards. The superior electric insulation properties of this material (about five orders of magnitude larger than that of the phenolic type) and much smaller water adsorption capacity are absolutely necessary when working with glass/membrane electrodes because of the high internal resistance of these devices.

The circuit design was also thoroughly considered in order to maintain the highest possible insulation between the different conductors. Care was also taken with regard to the soldering (all drops and spatter were carefully removed), and the pcb surface was subsequently washed with toluene to remove any traces of resin fluxes. These operations are essential to prevent the adsorption of water vapour on the polar/ionic components of the resin fluxes that may lead to consequent deterioration of the insulation properties of the pcb material.

The first type of pcb (pcb#1) contains opto-couplers, Telefunken type 4N26, which actuate directly the two burettes as well as a sound alarm installed to meet our purposes, i.e. signalling the end of each titration. The second type of pcb (pcb#2) contains the mechanical relay, a double pole double switch (DPDS) type Zettler AZ 820-2C-12DE, which allows the selection of the working electrode pair.

The Radiometer ABU 12 burettes are controlled manually either by pressing the front panel delivery button or through a hand-held button connected to an auxiliary port on the instrument. This port, also used to allow the remote control of the burette by the control unit, a Radiometer Titrator TTT60, was the mean chosen for the actuation of the burette. The simplicity of the electrical requirements underlying the delivery of titrant at a manually pre-selected speed, i.e. short-circuiting two pins on the port, allows the use of pcb#1 to directly control the burettes.

The alarm consists of a low-power electronic buzzer connected to the same power supply that feeds the relays and allows the non-attended operation of the device. This facility avoids undue time waste of the operator who is free to perform other tasks while the titration is on its way. Every time an end-point is reached, or at least the program judges it has reached it, the operator's attention is called upon thus allowing for the immediate set up of another run.

A commercial AC/DC power supply, Mercantile, capable of delivering 300 mA at 12 V was used as the power supply to actuate the relays and the alarm.

The selection of the working electrode pair, under computer control, usually suffers from noise transmitted from the computer to the measured signals. In order to achieve a good insulation between the digital noise generated inside the computer and the electrode potentials being measured, a two-step approach, schematically presented in figure 3, was chosen, i.e. the use of relays and two separated pcbs.

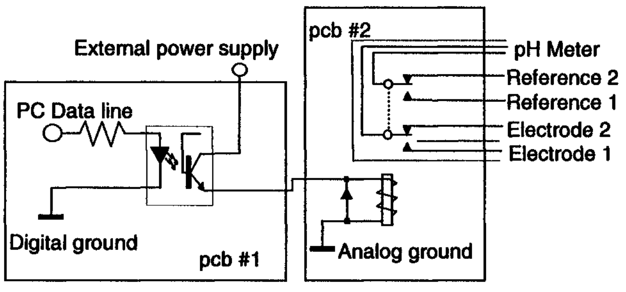

*Figure 3. Detailed electric diagram of the printed circuit boards (pcb#1 and #2).*

The DPDS relay configuration was chosen so as to maintain the same materials in the electrical connections of the circuit between the electrodes and the meter, therefore equilibrating all thermoelectric effects and maintaining the accuracy of the measurements. No special requirements were considered regarding the actuation time of the relay ($\leq$0.02 s), as the titration is a quite slow process in comparison to electronic timings, however, a time delay of 0.1 s was introduced in the data acquisition program. This time delay is essentially due to the pH meter's integration time (0.1 s) and should be adjusted accordingly whenever other pH meters are used or other systems are being measured.

The contact material, selected for the relays, was a nickel–silver alloy with 10 μm gold plating that assures a contact resistance lower than 10 mΩ, a reproducibility better than 10 mΩ and a thermoelectric potential of less than 1 μV. This last parameter being the primary concern when selecting the relays as the contact resistance becomes irrelevant in the face of the high internal resistance of the glass electrodes. It should be noted that in spite of the high quality of the materials, these relays are quite low priced, making the whole device rather inexpensive.

The first pcb (pcb#1) contains the opto-couplers, which isolates the computer electrical lines (the noisy ones) from the power used to actuate the relays. The second pcb (pcb#2) includes the mechanical relays, which further assures the insulation between the measured potentials and the command signal coming from pcb#1.

In order to minimize electrical leakage in the very high impedance circuit of the glass electrode, the electrical screening of this electrode was never interrupted, even when this electrode was not being measured. Furthermore, high-quality BNC connectors, Radiall 141410, were used (Teflon insulated), and the connecting wires between the panels and pcb#2 were all Teflon insulated (RS Components BS 2G 210 Type A).

### Conclusion

This type of switch is very simple and versatile, and can easily be expanded to be able to control four titration assemblies composed of burette and electrode pairs. Whenever burettes with digital control interfaces (RS-232, RS-422, RS-485, IEEE-488) are available, this device can be transformed into a nine-electrode pair switch, via the replication of the electronics described in figure 2.

The software control of the multi-electrode switch described is very simple, interactive and allows the choice of relay sequence, and is easily controlled in any high-level programming language (e.g. Basic) without performance degradation, thus allowing a rapid grasp and control by any inexperienced experimentalist.

The very low cost of the materials, the straightforward design and easiness of construction make this switch a very efficient and high-quality way of building multiple electrode measuring systems, thus constituting a convenient tool in automation procedures.

### Acknowledgment

We wish to thank Prof. Sousa Lopes, of the FCUL Physics Department, for graciously supplying the ADC8x12 converter.

### References

1. Zipper, J. J., Fleet, B. and Perone, S. P., *Analytical Chemistry*, **46** (1974), 2111.
2. Gaarenstroom, P. D., English, J. C., Perone, S. P. and Bixler, J. W., *Analytical Chemistry*, **50** (1978), 811.
3. Martin, C. R. and Freiser, H., *Analytical Chemistry*, **51** (1979), 803.
4. Beebe, K., Uerz, D., Sandifer, J. and Kowalski, B., *Analytical Chemistry*, **60** (1988), 66.
5. Lima, J. L. F. C., Montenegro, M. C. B. S. M., Alonso, J., Bartroli, J. and Raurichj, G., *Analytica Chimica Acta*, **234** (1990), 221.
6. Lapa, R. A. S. and Lima, J. L. F. C., *Journal of Automatic Chemistry*, **13** (1991), 119.
7. Spohn, U., Van der Pol, J., Eberhardt, R., Joksch, B. and Wandrey, Ch., *Analytica Chimica Acta*, **292** (1994), 281.
8. Frazer, J. W., Kray, A. M., Selig, W. and Lim, R., *Analytical Chemistry*, **47** (1975), 869.
9. Cunha, I. B. S. and Pasquini, C., *The Analyst*, **117** (1992), 905.
10. Santos, M. Soledade C. S. and Barbosa Ester, F. G., *7th International Conference on Surface and Colloid Science*, Vol. 1, Part 2, A4/ CL28, Compiègne (1991).
11. Santos M. Soledade, C. S., Seminar—Department of Chemistry, University of Lisbon (1995).

